# Recent Patents of Value to Dentists

**Published:** 1906-06

**Authors:** 


### RECENT PATENTS OF VALUE TO DENTISTS.

805,171, Artificial tooth, Emery L. Townsend, Los Angeles, California.
805,733, Tooth brush, Eduardo W. Key, Southwest City, Mo.
805,974, Manufacture of roof dental plates, Peter J. Malone, Altoona, Pa.

806,034, Artificial tooth fastening for bridgework, Thomas H. Whiteside, Youngstown, Ohio.

806,345, Suction attachment for dental plates, John A. Johnson, Middletown, Delaware.

806,300, Dental ligature applier, James Sorenson, Neenah, Wisconsin.

807,620, Mercury and amalgam separator, Henry J. Horstmann, Fort Wayne, Ind.

Copies of above patents may be obtained for ten cents each by addressing John A. Saul, Solicitor of Patents, Fendall Building, Washington, D. C.

